# Reverse micelle Extraction of Antibiotics using an Eco-friendly Sophorolipids Biosurfactant

**DOI:** 10.1038/s41598-017-18279-w

**Published:** 2018-01-11

**Authors:** Sing Chuong Chuo, Norfahana Abd-Talib, Siti Hamidah Mohd-Setapar, Hashim Hassan, Hasmida Mohd Nasir, Akil Ahmad, David Lokhat, Ghulam Md. Ashraf

**Affiliations:** 1 Faculty of Chemical and Energy Engineering, Universiti Teknologi Malaysia, 81310 UTM Skudai, Johor, Malaysia; 2 Centre of Lipids Engineering and Applied Research (CLEAR), Universiti Teknologi Malaysia, 81310 UTM Skudai, Johor, Malaysia; 3 SHE Empire Sdn. Bhd., No. 44, Jalan Pulai Ria 2, Bandar Baru Kangkar Pulai, 81300, Skudai, Johor Malaysia; 4 Department of Chemical Engineering, Howard College Campus, University of KwaZulu-Natal, Durban, 4041 South Africa; 5 King Fahd Medical Research Center, King Abdulaziz University, Jeddah, Saudi Arabia

## Abstract

Reverse micelles extraction of erythromycin and amoxicillin were carried out using the novel Sophorolipids biosurfactant. By replacing commonly used chemical surfactants with biosurfactant, reverse micelle extraction can be further improved in terms of environmental friendliness and sustainability. A central composite experimental design was used to investigate the effects of solution pH, KCl concentration, and sophorolipids concentration on the reverse micelle extraction of antibiotics. The most significant factor identified during the reverse micelle extraction of both antibiotics is the pH of aqueous solutions. Best forward extraction performance for erythromycin was found at feed phase pH of approximately 8.0 with low KCl and sophorolipids concentrations. Optimum recovery of erythromycin was obtained at stripping phase pH around 10.0 and with low KCl concentration. On the other hand, best forward extraction performance for amoxicillin was found at feed phase pH around 3.5 with low KCl concentration and high sophorolipids concentration. Optimum recovery of erythromycin was obtained at stripping phase pH around 6.0 with low KCl concentration. Both erythromycin and amoxicillin were found to be very sensitive toaqueous phase pH and can be easily degraded outside of their stable pH ranges.

## Introduction

Interest on antibiotics production had been growing since the introduction of germ theory of disease in the late 18^th^ century. In the year 2005, trade of antibiotics had reached 25 billion USD^1^, indicating that there is high demand of antibiotics in the world market. A large portion of antibiotics production cost comes from downstream processing due to the large volume but low antibiotics concentration product streams from the broth^2^. Filtration, solvent extraction, and crystallization are the classical steps for treating antibiotics product streams from broth^3^. However, the conventional liquid-liquid extraction method is often criticized for lack of options for solvents. Most solvents tested exhibited undesirable toxic properties or high solubility in water, making them unsuitable for extraction of antibiotics^4^. Butyl acetate is commonly used for liquid-liquid extraction of antibiotics but its high boiling point causes subsequent processing steps to be more expensive^5^. Another difficulty faced during conventional liquid-liquid extraction of antibiotics is the formation of a stable emulsion that significantly prolongs the extraction time and leads to possible degradation of antibiotics^6^.

Recently, an innovative liquid-liquid extraction method utilizing reversed micelles formed by surfactants had been studied for separation of proteins from aqueous solutions. The advantages of reverse micelle extraction include high selectivity, mild thermal operating conditions, low energy consumption, potential for large scale operation, and potential for continuous operation^7,8^. This extraction method had been tested for the extraction of different bio-molecules such as β-glucosidase^9^, chitosanases^10^, laccase^11^, lipase^12^, nattokinase^13^, penicillin G^14^, and polyphenol oxidase^15^. Modifications on the method such as using two surfactants to form the reverse micelles were also reported^16,17^.

Chemical surfactants such as bis(2-ethylhexyl) sodium sulfosuccinate (AOT), sodium dodecyl sulfate (SDS), and cetylmethylammonium bromide (CTAB) were always chosen by researchers to form their reverse micellar systems. On the other hand, biosurfactants were rarely investigated for the reverse micelle extraction of protein. In 2012, Peng *et al*.^11^ studied the potential of rhamnolipids biosurfactant to extract laccase produced by *C. versicolor*. Replacement of chemical surfactants with biosurfactant makes the operation to be more environmental friendly^18^. Besides that, the consumption of surfactant can be significantly reduced when using biosurfactant for reverse micelle extraction. However, studies regarding the potential use of biosurfactants for reverse micelle extraction of antibiotics have not yet been reported.

Besides rhamnolipids, sophorolipids are also one of the biosurfactants that is easy to obtain. They are produced from non-pathogenic yeast *Candida bombicola* ^19–21^. They can be in lactonic form or acidic form and are usually produced as a mixture of both. Sophorolipids are reported to be comparable or even better than chemical surfactants^22^. Therefore, they have the potential to be used as a sustainable and environmental friendly alternative for chemical surfactants.

In the present study, sophorolipids was used for the first time to form reverse micelles to extract antibiotics. Two commonly used antibiotics, erythromycin and amoxicillin, were chosen for the reverse micelle extraction. These two antibiotics are categorized in different antibiotic classes. They each possess their own characteristics and their behaviors during reverse micelle extraction are of interest. The main purpose of this study was to investigate the potential of sophorolipids as an alternative to chemical surfactants in reverse micelle extraction. This was done through investigating the effects of aqueous phase pH, ionic strength, and surfactant concentration on the reverse micelle extraction of antibiotics. Some insight on the optimum regions for the reverse micelle extraction was also provided.

## Result and Discussion

### Forward Extraction

In order to obtain models that can relate residual antibiotics concentration with the three manipulated variables, a full quadratic model was used as the starting model. Then, non-significant terms were eliminated based on ANOVA testing until the highest values of adjusted R^2^ were obtained. The significance level for the analysis is 0.05. In this way, a final reduced model was obtained for each set of experiment design. The terms included in the final reduced models and their estimated regression coefficient for forward extraction of erythromycin and amoxicillin are given in Tables 1 and 2, respectively. The p-values for each term included in the models are also shown. Box-Cox transformation with a lambda value of −4 was applied during the analysis of experimental data for erythromycin. The lack of fit for both models is not significant.

**Table 1:** Estimated regression coefficients for forward extraction of erythromycin.

Term	Coefficient	P-value
Constant	−6.13802	0.000
Feed phase pH	2.00568	0.000
KCl concentration	−0.01603	0.088
Sophorolipids concentration	−0.56852	0.433
Feed phase pH × Feed phase pH	−0.13529	0.005
Feed phase pH × KCl concentration	0.00127	0.151
KCl concentration × Sophorolipids concentration	0.00695	0.118

**Table 2:** Estimated regression coefficients for forward extraction of amoxicillin.

Term	Coefficient	P-value
Constant	−0.93786	0.000
Feed phase pH	1.31292	0.000
KCl concentration	0.01956	0.702
Sophorolipids concentration	−2.98886	0.013
Feed phase pH × Feed phase pH	−0.06319	0.253
Sophorolipids concentration × Sophorolipids concentration	2.17911	0.123
Feed phase pH × KCl concentration	−0.00400	0.019

**Figure 1:**
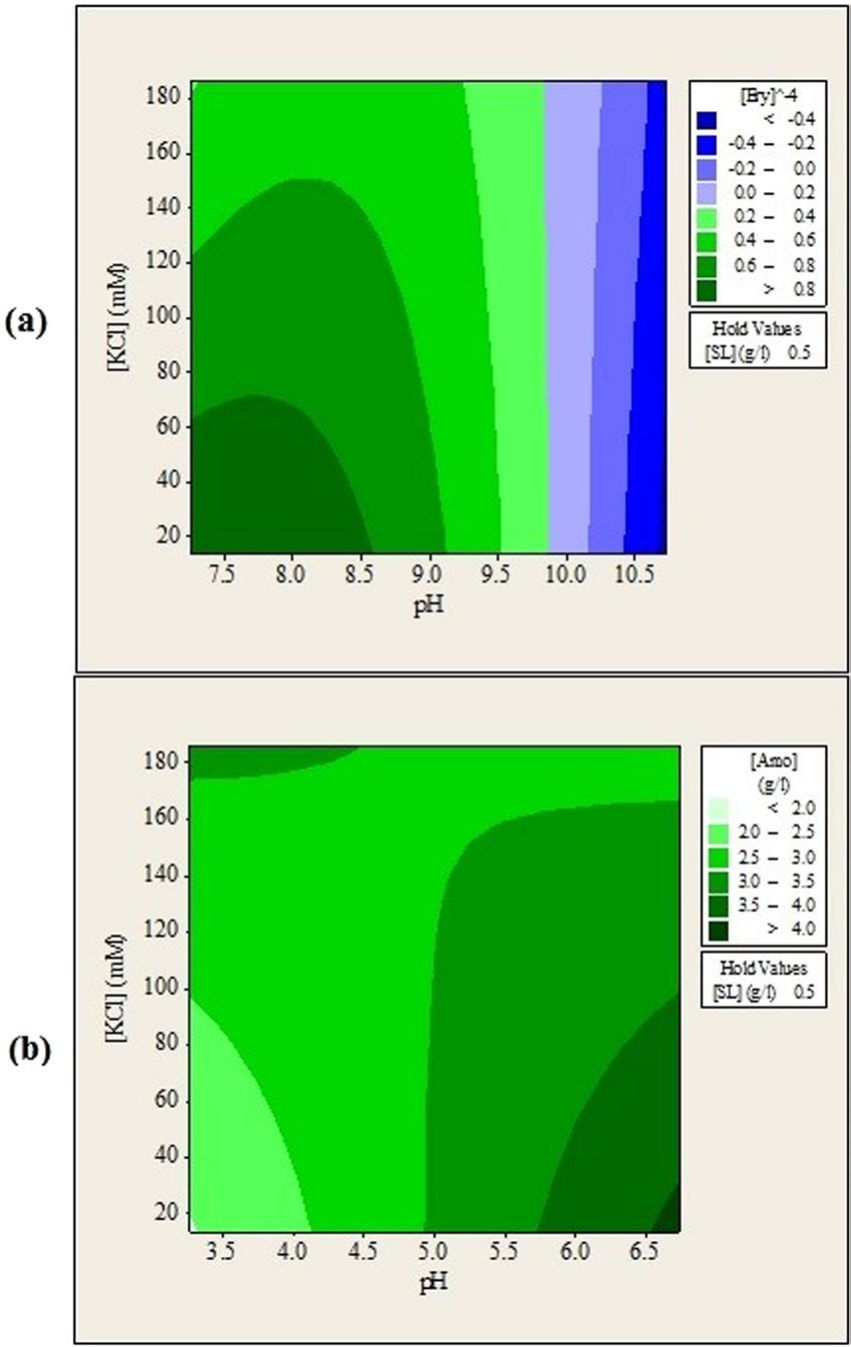
Contour plots of (**a**) erythromycin and (**b**) amoxicillin remained in aqueous solution (g/L) after forward extraction versus feed solution pH, KCl concentration.

**Figure 2:**
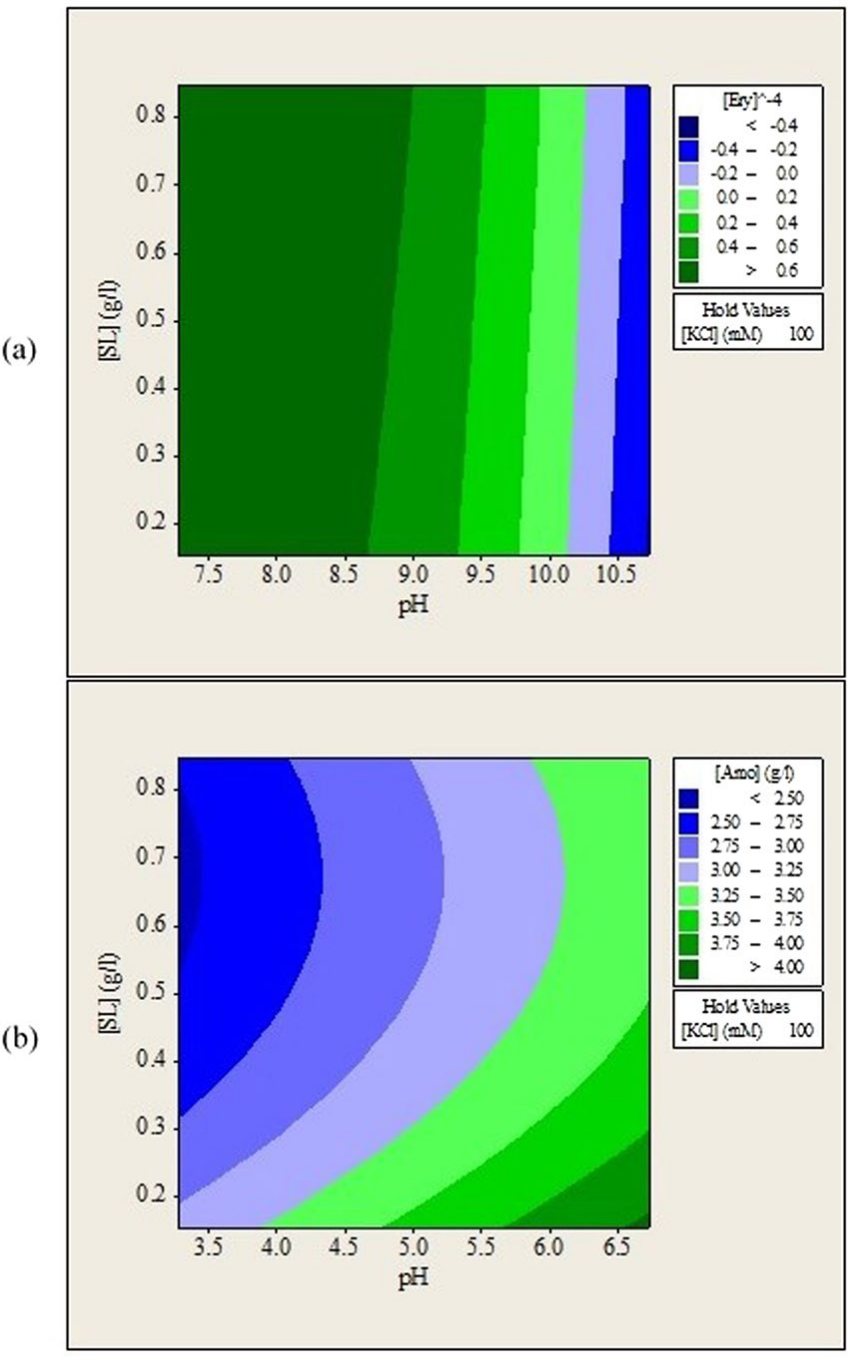
Contour plots of (**a**) erythromycin and (**b**) amoxicillin remained in aqueous solution (g/L) after forward extraction versus feed solution pH, sophorolipids concentration.

**Figure 3:**
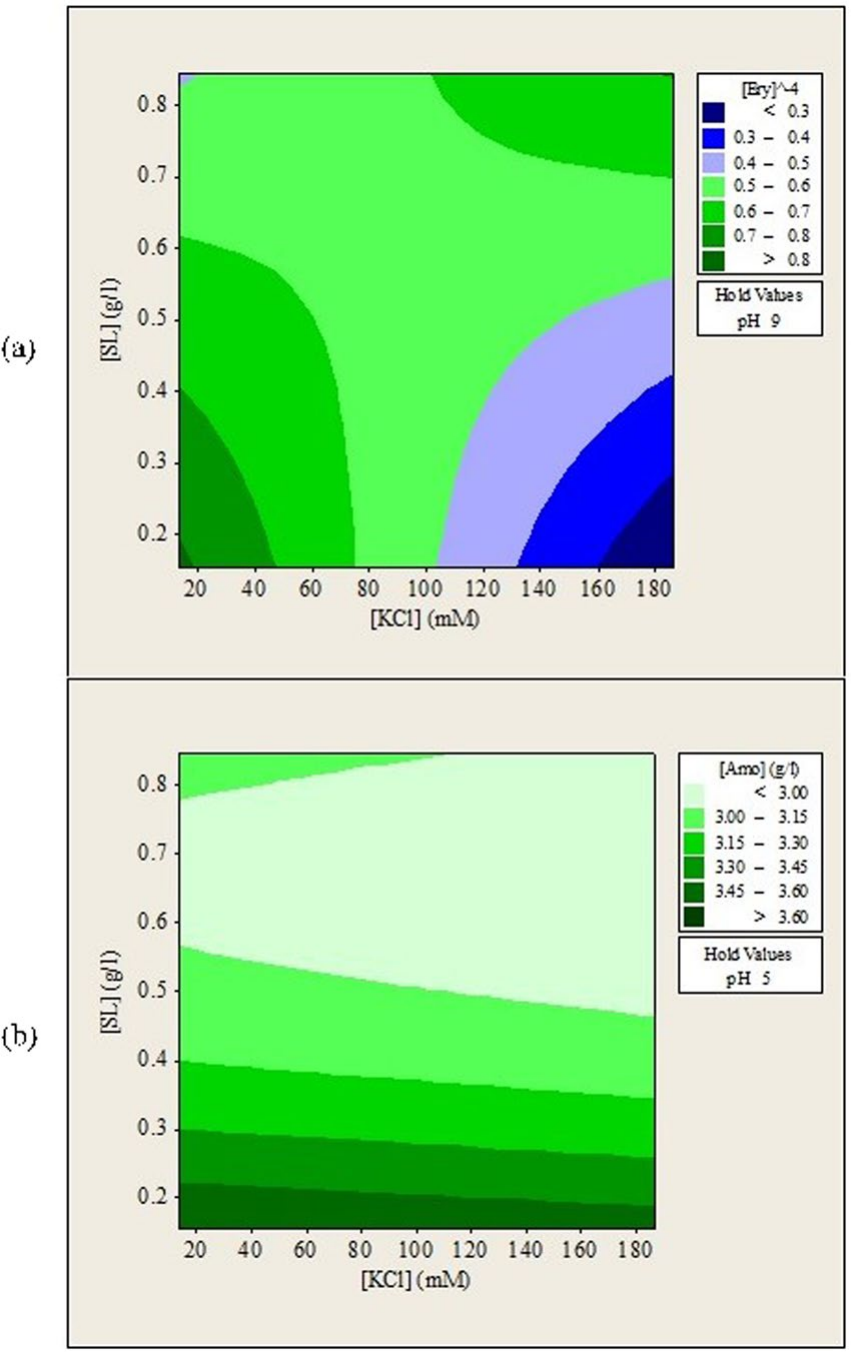
Contour plots of (**a**) erythromycin and (**b**) amoxicillin remained in aqueous solution (g/L) after forward extraction versus KCl concentration, sophorolipids concentration.

The experimental data reveals that aqueous phase pH is the most important factor during forward extraction of both antibiotics. This indicates that electrostatic interactions between antibiotics molecules and sophorolipids head groups are the main driving forces during the forward extraction. Although the effects of KCl salt and sophorolipids were not as significant as pH of solution, they were also essential to enable the forward extraction of erythromycin and amoxicillin. Without either of them, insignificant amount of antibiotics can be transferred into the isooctane organic phase. Figures 1, 2 and 3 show the contour plots for the forward extraction of erythromycin and amoxicillin. Each contour plot had a manipulated variable held at its middle value to investigate the effects of other two manipulated variables on the forward extraction of antibiotics.

### Effects of Aqueous Solution pH and Ionic Strength (KCl) on the Forward Extraction of Erythromycin and Amoxicillin

The forward extraction of erythromycin was highest when pH of feed solution was near 8.0 and deceased when the pH was increased from 8.5 to 10.0 as shown in Figs 1 and 2. Erythromycin were reported to be most stable between pH 7.0 and 9.0^5^. Erythromycin will be degraded when solution pH becomes less than 6.0 or higher than 10.0^23,24^. In this study, optimum extraction of erythromycin was found located at feed solution pH between 7.27 and8.5 where erythromycin is most stable.

Besides being stable, erythromycin also has a net positive charge when pH of feed solution is kept below 8.6^25^. This allows strong attractive electrostatic interactions to occur between erythromycin and sophorolipids head groups thus more erythromycin can be extracted. Erythromycin changed predominantly to its non-polar form when the pH of the feed solution was increased to above 8.6. This caused the attractive electrostatic interactions to decrease and led to reduction of erythromycin been extracted at feed solution pH 9.0 and 10.0. Likewise, forward extraction of amoxicillin was highest when pH of feed solution is less than 4.0 and it deceased when pH of feed solution was increased from 4.0 to 6.7 as shown in Figs 1 and 2. This is because when pH of feed solution is lower than isoelectric point of amoxicillin (pI = 4.7), amoxicillin will have net positive charge and contributes to stronger attractive electrostatic interactions between amoxicillin and sophorolipids head group. At solution pH higher than 4.7, amoxicillin will have net negative charge and the electrostatic interactions become repulsive forces thus reducing the forward extraction of amoxicillin. Generally, solution pH is adjusted so that the molecules have opposite charge as the surfactant used to ensure extraction occurs. The optimum pH for forward extraction of lipase using AOT reverse micellar system was found to be at 6.5, which is lower than pI of lipase at 8.2^12^. Extraction of β-glucosidase using mixed reversed micelle system showed optimum forward extraction pH slightly lower that pI^9^. Similar trends were also reported during the reverse micelle extraction of penicillin G^14^. This indicates that electrostatic interactions are the most important forces during forward extraction. Similar trends were also reported during the reverse micelle extraction of penicillin G^26^, lipase^12^, and β-glucosidase^9^.

Figure 1 shows a complex trend from the combined effects of feed solution pH and KCl concentration on the forward extraction of amoxicillin. Investigation on the interactions between variables is important to understand the behavior of the extraction system in detail. This can avoid the situations where the benefits of varying a variable are offset due to its inherent interactions with other variables. The effects of these interactions can be identified easily with design of experiment and statistical analysis. Figure 2 shows that at pH lower than 4.5, extraction of amoxicillin was reduced when KCl concentration was increased. On the other hand, extraction of amoxicillin was enhanced when KCl concentration was increased at pH higher than 5.0. This observation was probably caused by the shielding effects of KCl salt. When solution pH is higher than 4.7, the resulting electrostatic interactions between amoxicillin and sophorolipids were repulsive. Therefore, higher amount of KCl helped to shield the repulsive forces and allowed more amoxicillin to be extracted. However, high concentration of KCl causes reverse effects when solution pH is lower than 4.7 because the attractive forces between amoxicillin and sophorolipids are shielded. For extraction of erythromycin, the optimum region was observed at solution pH lower than 8.5 and KCl concentration lower than 60 mM. No complex trend was found suggesting that erythromycin and amoxicillin have different ways to interact with the sophorolipids reverse micelles.

### Effects of Aqueous Solution pH and Sophorolipids Concentration on the Forward Extraction of Erythromycin and Amoxicillin

Figure 2 show that varying sophorolipids concentration did not have significant effects on the forward extraction of erythromycin as long as pH of solution was kept below 8.5. Increasing sophorolipids concentration from 0.15 to 0.84 g/L only increased erythromycin extraction slightly. Sophorolipids concentration between 0.6 and 0.8 g/L favored the forward extraction of amoxicillin in all feed solution pH investigated. Increasing the concentration of sophorolipids increased the number of functional reverse micelles and led to better forward extraction of antibiotics. However, forward extraction of amoxicillin decreased when sophorolipids concentration was further increased to above 0.8 g/L. This was due to the micellar clustering^27^ and intermicellar collisions^11^ that occur at high surfactant concentration. Some researchers reported that increasing surfactant concentration will no longer enhance forward extraction when certain surfactant concentrations were reached and the amount of impurities extracted were increased instead^27,28^. This observation suggests that high concentration of surfactant should be avoided during reverse micelle extraction. No complex trend was observed from Fig. 2 but the different shape of contour plots indicate that erythromycin was more easily encapsulated in sophorolipids reverse micelles as compared to amoxicillin.

### Effects of Ionic Strength (KCl) and Sophorolipids Concentration on the Forward Extraction of Erythromycin and Amoxicillin

The combined effects of KCl concentration and sophorolipids concentration on forward extraction of erythromycin were complex as shown in Fig. 3. More erythromycin was extracted at KCl concentration lower than 75 mM and sophorolipids concentration lower than 0.6 g/L or at KCl concentration higher than 100 mM and sophorolipids concentration higher than 0.7 g/L. This can be related to the roles of KCl salt in forming functional reverse micelles for the extraction process. KCl helps to shield the repulsive forces between surfactant head groups so that the surfactant molecules can come closer together to form stable and functional reverse micelles. The experiment showed that low concentration of KCl was sufficient to form functional reverse micelles when low sophorolipids concentration was used. Higher concentration of KCl was needed when high concentration of sophorolipids was used. The presence of functional sophorolipids reverse micelles determined the forward extraction of erythromycin. On the other hand, sophorolipids concentration between 0.6 and 0.8 g/L favored the forward extraction of amoxicillin for all KCl concentration tested. The observations showed that structures of both antibiotics had their impacts on the formation of sophorolipids reverse micelles.

### Backward Extraction

Backward extraction is generally considered as more difficult than forward extraction because the bonds formed within reverse micelles need to be broken such that solutes can be recovered. The solutes can also be salted out by reducing the size of reverse micelles but doing this may cause irreversible damage to the solutes. Sometimes a second salt or co-solvent such as alcohols is added during backward extraction to increase the recovery of solutes^11^.The analysis procedures used in the forward extraction were applied also for the backward extraction experimental data except that the Box-Cox transformation was not required. A final reduced model was obtained for each set of experiment design. The terms included in the final reduced model and their estimated regression coefficients for backward extraction of erythromycin and amoxicillin were given in Tables 3 and 4. The lack of fit for both models is not significant.

**Table 3:** Estimated regression coefficients for backward extraction of erythromycin.

Term	Coefficient	P-value
Constant	7.70088	0.000
Stripping phase pH	−1.69015	0.000
KCl concentration	−9.57829E-05	0.709
Sophorolipids concentration	−0.98445	0.175
Stripping phase pH × Stripping phase pH	0.10671	0.000
Stripping phase pH × Sophorolipids concentration	0.11938	0.173

**Table 4:** Estimated regression coefficients for backward extraction of amoxicillin.

Term	Coefficient	P-value
Constant	0.40510	0.000
Stripping phase pH	0.08486	0.606
KCl concentration	0.00036	0.149
Sophorolipids concentration	−1.61076	0.004
Stripping phase pH × Stripping phase pH	−0.00761	0.525
KCl concentration × KCl concentration	−3.71412E-06	0.440
Sophorolipids concentration × Sophorolipids concentration	1.39672	0.000

**Figure 4:**
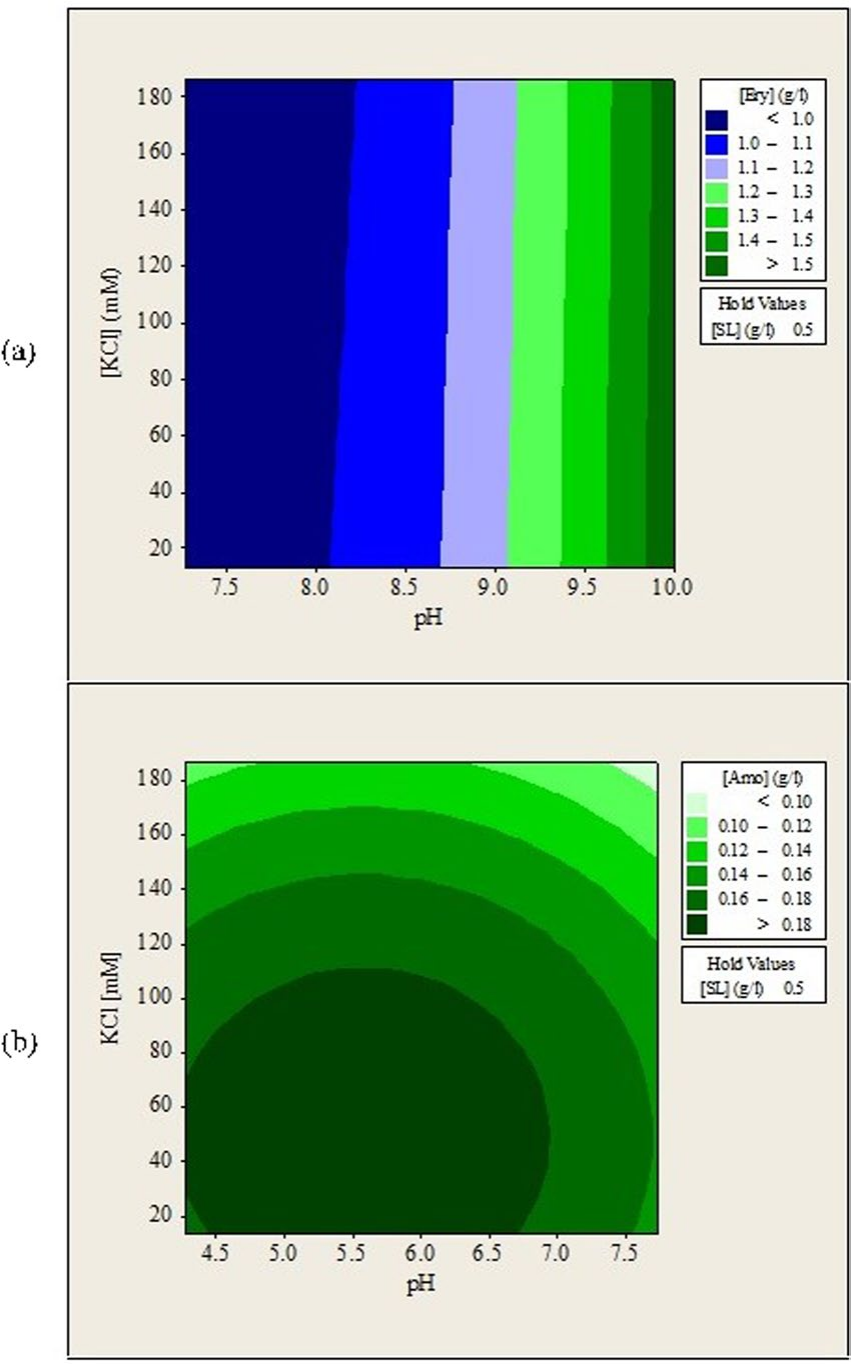
Contour plots of (**a**) erythromycin and (**b**) amoxicillin recovered in aqueous solution (g/L) after backward extraction versus stripping solution pH, KCl concentration.

**Figure 5:**
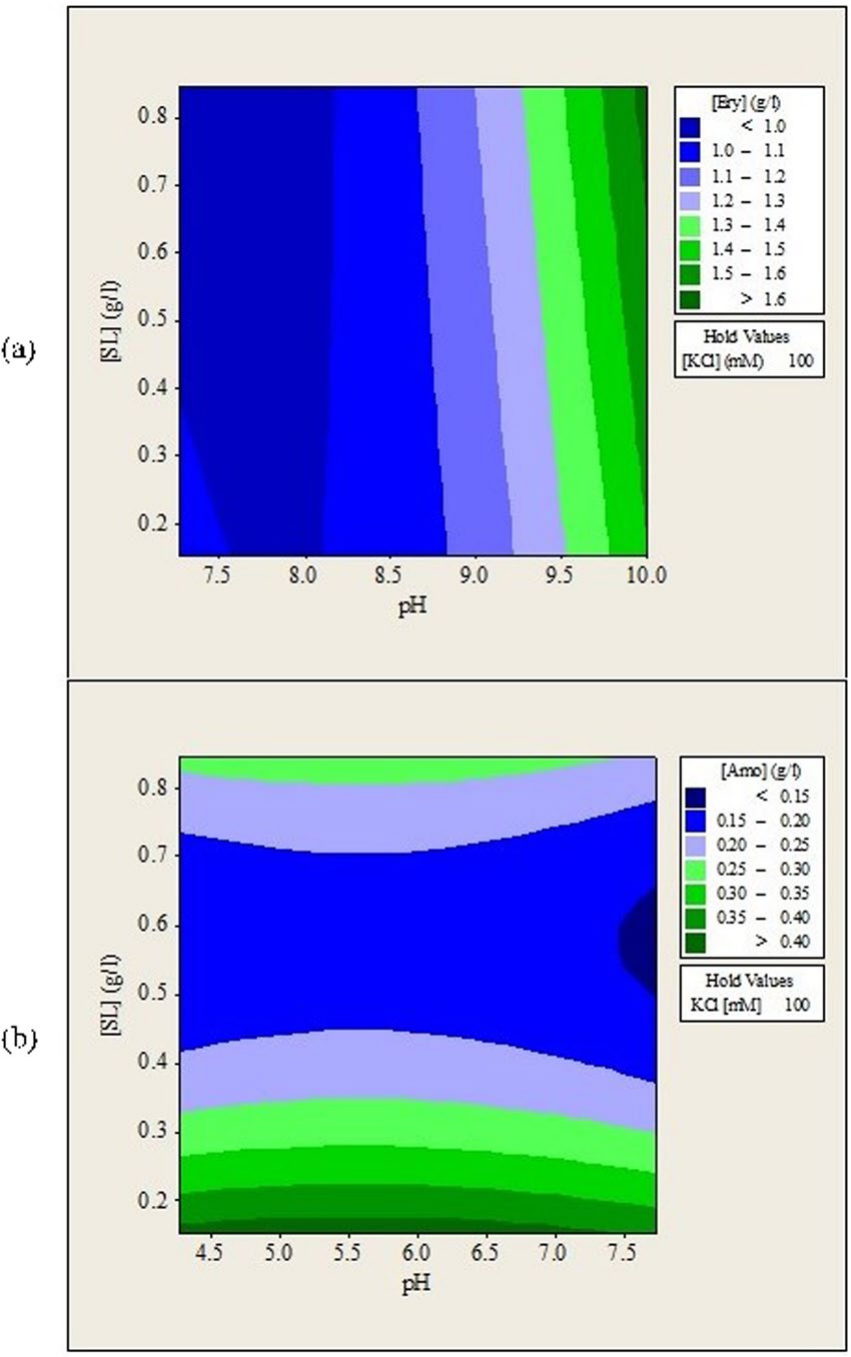
Contour plots of (**a**) erythromycin and (**b**) amoxicillin recovered in aqueous solution (g/L) after backward extraction versus stripping solution pH, sophorolipids concentration.

**Figure 6:**
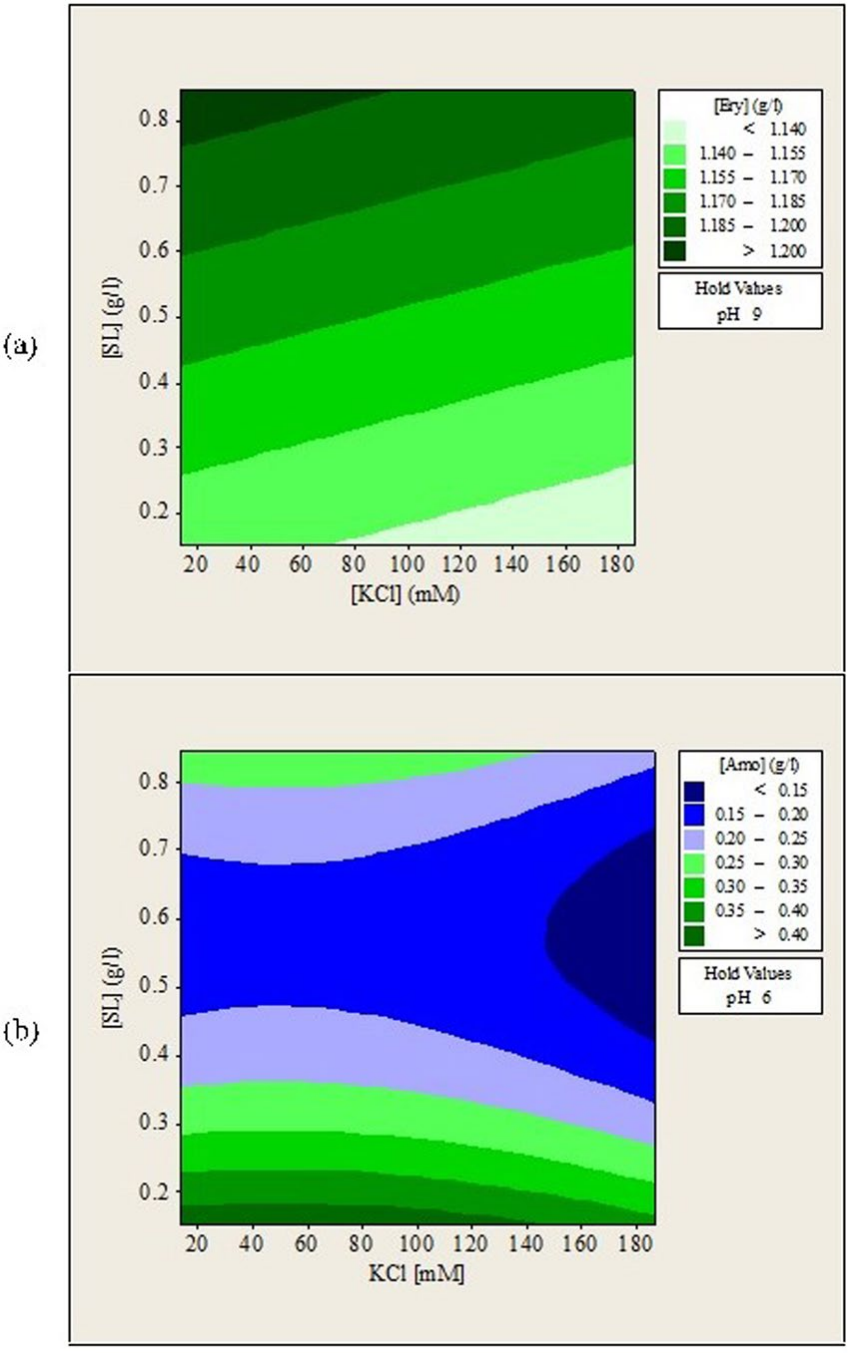
Contour plots of (**a**) erythromycin and (**b**) amoxicillin recovered in aqueous solution (g/L) after backward extraction versus KCl concentration, sophorolipids concentration.

The pH of the stripping solution is the most significant factor for backward extraction of erythromycin while sophorolipids concentration is the most significant factor for backward extraction of amoxicillin. The effects of sophorolipids concentration were also found to be statistically significant during forward extraction of amoxicillin. These observations suggested that both antibiotics have different interactions with sophorolipids reverse micelles. Figures 4, 5 and 6 show the contour plots for the backward extraction of erythromycin and amoxicillin. Each contour plot had a manipulated variable held at its middle value to investigate the effects of other two manipulated variables on the backward extraction of antibiotics.

### Effects of Aqueous Solution pH and Ionic Strength (KCl) on the Backward Extraction of Erythromycin and Amoxicillin

Figure 4 shows that maximum recovery of erythromycin was obtained when pH of aqueous solution was near 10.0 regardless of KCl concentration. Since erythromycin is in non-polar form when solution pH is higher than 8.6, the electrostatic interactions between erythromycin and sophorolipids head group are weaker. This leads to the release of erythromycin into the aqueous phase. This observation showed that the main attractive forces that encapsulate erythromycin inside sophorolipids reverse micelles are the electrostatic interactions. In a backward extraction study of tannase and lipase, maximum recovery was also obtained at solution pH where electrostatic interactions were the minimum^27^. However, when backward extraction was conducted at solution pH = 10.7, very low concentration of erythromycin could be detected in the aqueous solution due to most of them been degraded at extreme pH conditions.

Figure 4 shows that an optimum region for amoxicillin recovery existed at solution pH 4.5–6.5 and KCl concentration lower that 100 mM. When the pH of aqueous solution was near or above 4.7, the attractive interactions between amoxicillin and sophorolipids reverse micelles became weaker because they both had the same surface charges. This phenomenon contributed to the release of amoxicillin from sophorolipids reverse micelles into the aqueous solutions. Amoxicillin recovery decreased when pH of solution was increased to above 7.0. White precipitates were observed at alkaline conditions, which suggested that amoxicillin had denatured. Our previous study also reported similar observations^29^. Amoxicillin was reported to be most stable when pH of solution is kept near 6.0^30^ and degradation of amoxicillin starts to occur at a faster rate when the solution pH is higher than 7.0^31^. Both antibiotics showed their sensitivity towards pH change. Therefore, solution pH must be adjusted carefully to avoid degradation of antibiotics.

Figure 4 also shows that the recovery of erythromycin and amoxicillin was maximized at KCl concentration as low as 20 mM. Reduction of both antibiotics recovery with increasing KCl concentration was displayed. The observations were most probably caused by the shielding effects of KCl salt around sophorolipids reverse micelles. It was also possible that antibiotics were deformed when been salted out from sophorolipids reverse micelles. Reduction of protein recovery at high salt concentration during backward extraction was also reported in other studies^12,28^. No complex trend was observed in Fig. 4.

### Effects of Aqueous Solution pH and Sophorolipids Concentration on the Backward Extraction of Erythromycin and Amoxicillin

Figure 5 shows that maximum recovery of erythromycin occurred at solution pH near 10.0 and increasing sophorolipids concentration increased erythromycin recovery slightly. This was directly related to the higher amount of erythromycin extracted during forward extraction at higher sophorolipids concentration. Higher erythromycin concentration in the organic phase led to more erythromycin been released into the aqueous solution during backward extraction.

Highest amoxicillin recovery was observed at sophorolipids concentration less than 0.2 g/L, which was unusually low within all solution pH, tested. However, there were large differences among the recorded experimental values of amoxicillin concentration when replicates were conducted at 0.154 g/L sophorolipids concentration. It is a region where amoxicillin recovery is very unstable. This observation suggested that reverse micelles formed when sophorolipids concentration is less than 0.2 g/L are relatively unstable compared to those formed at higher sophorolipids concentration such as between 0.4 and 0.7 g/L. Destabilization of reverse micelles within the organic phase led to direct contact of amoxicillin with isooctane, which in turn led to degradation of amoxicillin. On the other hand, destabilization of reverse micelles near interfaces allows more amoxicillin to be released into the stripping phase. Sophorolipids concentration less than 0.2 g/L should not be used for the reverse micelle extraction of amoxicillin due to the highly inconsistent recovery observed. No complex trend was observed from Fig. 5 but the different shape of contour plots indicate that each antibiotic may had interacted with sophorolipids reverse micelles in a different manner.

### Effects of Ionic Strength (KCl) and Sophorolipids Concentration on the Backward Extraction of Erythromycin and Amoxicillin

Optimum erythromycin recovery was observed at KCl concentration lower than 60 mM and sophorolipids concentration higher than 0.75 g/L as shown in Fig. 6. Nevertheless, the difference between highest and lowest erythromycin concentration recovered was only 0.06 g/L. This indicates that effects of KCl concentration and sophorolipids concentration were less significant than effects of solution pH alone. Amoxicillin recovery was maximized at sophorolipids concentration less than 0.2 g/L for all KCl concentration tested but the unstable reverse micelles make this experiment conditions unreliable. Reduction of amoxicillin recovery when sophorolipids concentration was increased to near 0.6 g/L was observed as shown in Figs 5 and 6. Amoxicillin forward extraction was maximized at sophorolipids concentration near 0.6 g/L, indicating that partitioning of amoxicillin into sophorolipids reverse micelles was favored at this concentration. Therefore, it was hard for amoxicillin to be released into the aqueous phase during backward extraction and led to lower recovery.

### Discussions

Varying pH of aqueous solution has significant effects on reverse micelle extraction of both erythromycin and amoxicillin. This indicates that electrostatic interactions are the most important forces during the extraction process. Both antibiotics are having net negative surface charge when solution pH is lower than their pI values, increasing their attractive electrostatic interactions with sophorolipids head group and favor forward extraction process. On the other hand, solution pH higher than their pI values causes the attractive forces to diminish due to the change in their net surface charges and favor the backward extraction process. Therefore a similar trend was observed for reverse micelle extraction of both antibiotics when solution pH is varied.

Main purpose of KCl salt is to help in the formation of functional sophorolipids reverse micelles. Low concentration of KCl was found to be sufficient for reverse micelle extraction of erythromycin and amoxicillin. The increasing shielding effect and micellar size reduction effect at higher concentration of KCl will hinder the extraction of antibiotics. Similar trend was observed for reverse micelle extraction of both antibiotics when KCl concentration is varied.

Erythromycin and amoxicillin have negligible solubility in isooctane. The extraction of both antibiotics was made possible due to the addition of sophorolipids reverse micelles. More reverse micelles available at higher concentration of sophorolipids allow more antibiotics to be extracted. The effects of varying sophorolipids concentration were more pronounced during the reverse micelle extraction of amoxicillin. However, micellar clustering and deformation of micellar shape that occurs at high surfactant concentration may hinder the extraction process.

Extraction results showed that sophorolipids reverse micellar system is better for the extraction of erythromycin as compared to amoxicillin. The concentration of erythromycin been extracted and recovered is considerably higher than those of amoxicillin. The differences in molecular structures and nature of the antibiotics may have caused the difference in extraction efficiency. This indicates that erythromycin and amoxicillin may have different interactions with sophorolipids reverse micelles. The optimum regions for reverse micelle extraction of both antibiotics based on this study are given in Table 5.

**Table 5:** Optimum regions for reverse micelle extraction of amoxicillin and erythromycin.

Antibiotics	Extraction	pH	KCl concentration (mM)	Sophorolipids concentration (g/L)
Amoxicillin	Forward	3.3–3.5	13.5–50.0	0.6–0.8
	Backward	5.5–6.0	50.0	0.2
Erythromycin	Forward	7.0–8.0	13.5–50.0	0.2–0.4
	Backward	9.0–10.0	13.5–100.0	0.6–0.8

**Figure 7:**
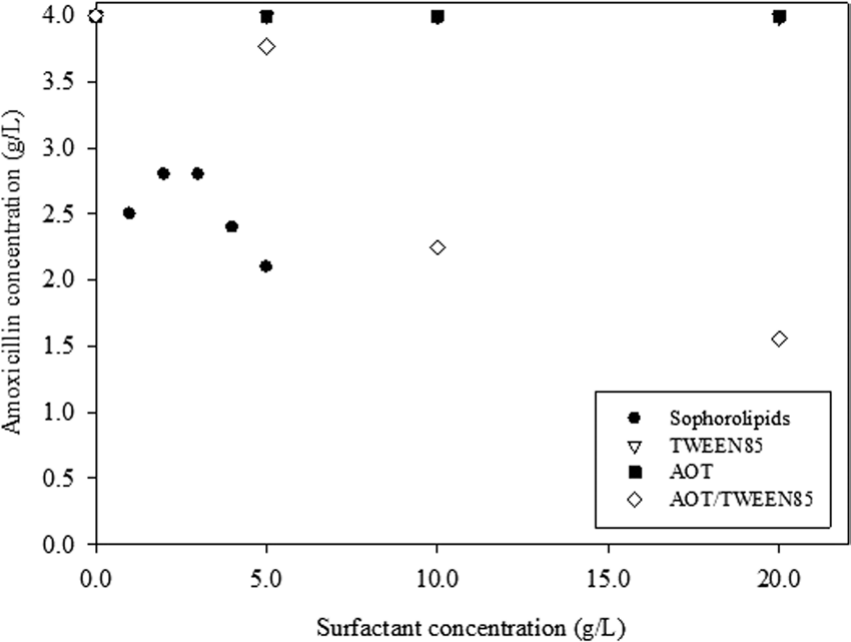
Forward extraction of amoxicillin using different reverse micellar system.

Figure 7 shows the comparison between sophorolipids, AOT, and TWEEN 85 reverse micellar system for the forward extraction of amoxicillin. The results for AOT and TWEEN 85 surfactants were from our previous study^29^. Significant forward extraction of amoxicillin was achieved at much lower surfactant concentration when sophorolipids was used as compared to other surfactants. Study by Peng *et al*.^11^ also showed that low concentration of rhamnolipids biosurfactant (2.15 g/L) was optimum for the extraction of laccase. Other studies using chemical surfactants needed higher amount of surfactants in order to achieve significant extraction of protein, such as 11.11 g/L AOT for lipase extraction^12^, 14.58 g/L AOT for bovine serum albumin extraction^32^, 18.22 g/L for nattokinase extraction^13^, 88.91 g/L for β-galactosidase extraction^33^, or 7.29 g/L CTAB for tannase extraction^27^. By using biosurfactants to replace chemical surfactants in reverse micelle extraction, the amount of surfactants can be greatly reduced.

Conventionally, extraction of antibiotics is carried out through liquid-liquid extraction using butyl acetate. However, it takes as long as 35 h for phase separation after the extraction process due to the formation of stable emulsion^34^. In our study, the mixture only took few minutes to separate into two clear distinct phases after stirring. High boiling point of butyl acetate also causes subsequent antibiotic recovery process to be more costly^5^. On the other hand, recovery of antibiotics during reverse micelle extraction is conducted easily by mixing the organic phase with aqueous stripping phase without the need of extra equipment. The choices of organic solvent for conventional liquid-liquid extraction are very limited due to their high solubility in aqueous or high toxicity^4^. By using reverse micelle extraction, more choices of organic solvents are available because the transfer of antibiotics between phases is enabled by reverse micelles. Furthermore, the encapsulation of antibiotics inside reverse micelles helps to protect the antibiotics from degradation through direct contact with organic solvents, thus increasing the final product yield. In this study, we went one step further by replacing the synthetic surfactants that are widely studied in reverse micelle extraction with biosurfactant to ensure that the extraction process is sustainable and environmental friendly.

Besides organic solvents, ionic liquids were also studied for extraction of antibiotics^35^. However, it has difficulty in recovering antibiotics from the ionic liquids. High pressure CO_2_ was proposed for recovery of antibiotics from ionic liquids but this will greatly increase the costs and there are still several unsolved uncertainties^5^. Another technique that had been studied for separation of antibiotics is filtration, including nanofiltration and ultrafiltration. Formation of stable emulsion that hinders phase separation was still observed^2^ and the large volume of process stream from filtration will lead to longer time and higher cost in subsequent processing^36^. Filtration technique is more suitable for removal of antibiotics from wastewater^37^. Adsorption technique is also used for separation of antibiotics. Its applications are focused on high resolution separation such as for high performance liquid chromatography. It has difficulty to processing high volume stream as the processing time will be very long and there is the risk of antibiotics degradation during the extraction process^38^. By using reverse micelle extraction, the extraction and recovery of antibiotics can be carried out easily without formation of stable emulsion. It is suitable for processing high volume process stream^15^. Application of biosurfactant also allows greener extraction process of antibiotics to be conducted.

## Conclusions

Sophorolipids reverse micellar system was tested for the reverse micelle extraction of erythromycin and amoxicillin. Extraction and recovery of antibiotics were affected by solution pH, ionic strength, and sophorolipids concentration. The pH of aqueous solutions is the most significant factor during reverse micelle extraction of both antibiotics. This suggests that electrostatic interactions between antibiotic and sophorolipids head groups controlled the extraction process. Concentration of sophorolipids is also a significant factor during reverse micelle extraction of amoxicillin. Although the effects of KCl concentration seem less significant, it is necessary for the formation of functional reverse micelles. Optimum conditions for reverse micelle extraction of erythromycin and amoxicillin were identified through contour plots. Replacing chemical surfactants with sophorolipids can render the reverse micelle extraction process more environmental friendly and sustainable. Sophorolipids also significantly reduce the amount of surfactant needed during the reverse micelle extraction.

### Material

Pure erythromycin A (C_37_H_67_NO_13_, 733.94 g/mol) and amoxicillin trihydrate (C_16_H_19_N_3_O_5_S.3H_2_O, 419.45 g/mol) were purchased from bio-WORLD, USA. Sophorolipids, potassium chloride salt (KCl), Hydrochloric acid (HCl), sodium hydroxide (NaOH), and isooctane were purchased from Sigma-Aldrich, USA. All materials were of analytical grade and used without further purification. Demineralized water (DMW) and isooctane were used to prepare the aqueous and organic phases throughout the study. The pH of aqueous solutions was prepared using HCl and NaOH for pH range 3–11. KCl salt was used to adjust the ionic strength of aqueous solutions. Desired amount of erythromycin or amoxicillin were dissolved in the aqueous solutions to obtain feed solutions. Isooctane was used to make the reverse micellar phase by dissolving desired amount of sophorolipids in it.

Aqueous solutions with pH ranges 3–11 were prepared using demineralized water and HCl/NaOH solutions. Various studies reported the production of amoxicillin at around 4 g/L within 5 h of operation^39,40^. Since reverse micelle extraction is a fast process and able to process that amount of antibiotics within 5 h, thus 4 g/L was chosen as the starting antibiotic concentration for this study. 4.0 g/L of erythromycin or amoxicillin and 13.5 mM–186.5 mM of KCl were dissolved in the aqueous phase to obtain the feed phase for forward extraction. 15.5 mM–200 mM of KCl were dissolved in the aqueous phase to obtain the stripping phase for backward extraction. 0.15 g/L–0.85 g/L of sophorolipids were dissolved in isooctane to obtain the reverse micellar phase for the reverse micellar phases were prepared right before experiment to prevent solvent loss through vaporization.

**Figure 8:**
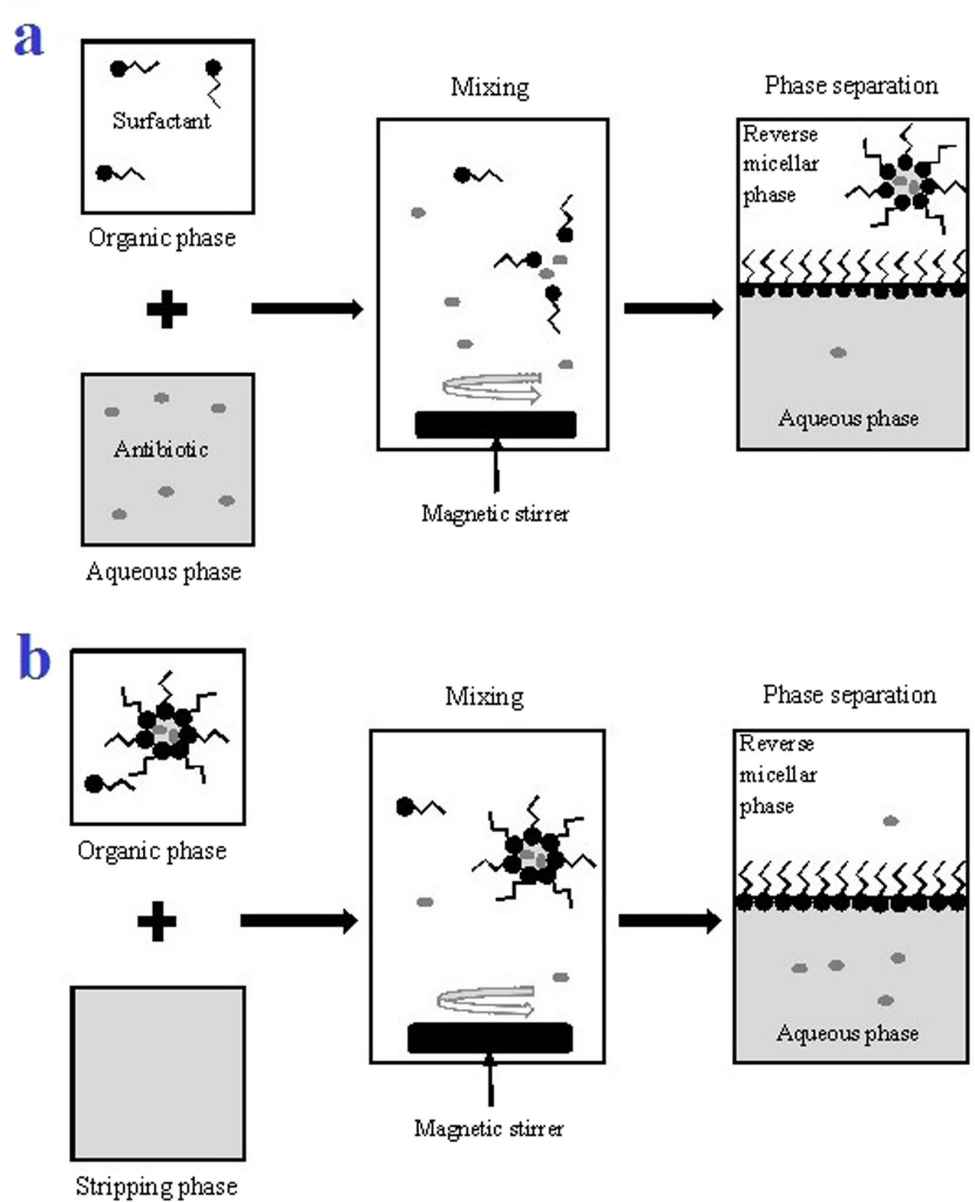
Schematic interaction of antibiotics and surfactants (**a**) forward extraction and (**b**) backward extraction.

After preparing the standard solutions, reverse micelle extraction of erythromycin and amoxicillin were conducted. All the extractions were carried out at room temperature (27 °C) and atmospheric pressure (1 atm). During forward extraction, 5 mL of feed aqueous phase were mixed with 5 mL of organic phase by stirring at 200 rpm for 30 min. Then, the mixtures were left for phase separation by gravity action. The mixture separated into two clear and distinct phases within an hour after stirring. The residual antibiotic concentration in the aqueous phase was measured though UV spectrophotometer. During backward extraction, the organic phases obtained from forward extraction were mixed with equal volume of stripping aqueous phases by stirring at 200 rpm for 30 min. Then, the mixtures were left for phase separation by gravity action. The reverse micelle extraction (forward and backward) with interaction of surfactant with antibiotics are described in Fig. 8. After two clear and distinct phases were achieved, the residual erythromycin and amoxicillin in aqueous solutions was measured using UV spectrophotometer.

The effects of aqueous phase pH, sophorolipids concentration, and KCl concentration in aqueous phases were investigated throughout the experiment. Some preliminary experiments consisting trial and error runs were conducted to test the potential of sophorolipids to extract antibiotics and to obtain starting points for subsequent experiments. This study was mainly conducted by applying central composite design for the reverse micelle extraction of erythromycin and amoxicillin. By utilizing this design of experiment, more thorough information such as significance of factors and interaction effects between factors can be obtained through statistical analysis. Wider view on the trend of the extraction results can be obtained in the form of surface plots and the optimum regions can be identified. Furthermore, all this information can be obtained through significantly less number of experiment runs compared to classical one-factor-at-a-time experimental procedure.

The main response during the reverse micelle extraction was the amount of erythromycin or amoxicillin remaining in aqueous phase after extraction was conducted, and the manipulated variables were aqueous phase pH, sophorolipids concentration, and KCl concentration. Tables 6 and 7 show the lower and upper values of the manipulated variables used during the reverse micelle extraction of erythromycin and amoxicillin respectively. All values are in uncoded form. The value of α for the central composite design was √3. Six center points were included for each set of experiment. The experimental data was analyzed using Minitab 16.

**Table 6:** Lower and upper values for erythromycin experiment.

Variables	Forward extraction		Backward extraction	
	Lower value	Upper value	Lower value	Upper value
pH	8.0	10.0	8.0	10.0
Sophorolipids concentration (g/l)	0.30	0.70	0.30	0.70
KCl concentration (mM)	50.0	150.0	50.0	150.0

**Table 7:** Lower and upper values for amoxicillin experiment.

Variables	Forward extraction		Backward extraction	
	Lower value	Upper value	Lower value	Upper value
pH	4.0	6.0	5.0	7.0
Sophorolipids concentration (g/l)	0.30	0.70	0.30	0.70
KCl concentration (mM)	50.0	150.0	50.0	150.0
